# Effects of flaxseed supplementation on weight loss, lipid profiles, glucose, and high‐sensitivity C‐reactive protein in patients with coronary artery disease: A systematic review and meta‐analysis of randomized controlled trials

**DOI:** 10.1002/clc.24211

**Published:** 2024-01-16

**Authors:** Hamid Reza Sabet, Mohammad Ahmadi, Mehdi Akrami, Mahsa Motamed, Omid Keshavarzian, Mozhan Abdollahi, Mehdi Rezaei, Hamed Akbari

**Affiliations:** ^1^ Medical Journalism Department, School of Paramedical Sciences Shiraz University of Medical Sciences Shiraz Iran; ^2^ Students' Scientific Research Center Tehran University of Medical Sciences Tehran Iran; ^3^ Cardiovascular Department Shiraz University of Medical Sciences Shiraz Iran; ^4^ Department of Psychiatry Tehran University of Medical Sciences Tehran Iran; ^5^ Shiraz School for Medicine Shiraz University of Medical Sciences Shiraz Iran; ^6^ Student Research Committee, School of Medicine Shiraz University of Medical Sciences Shiraz Iran; ^7^ Department of Cardiology, Fars‐Iranian Heart Association Fars Society of Internal Medicine Shiraz Iran; ^8^ Department of Clinical Biochemistry, Faculty of Medicine Kerman University of Medical Sciences Kerman Iran

**Keywords:** anthropometric indices, coronary artery diseases, flaxseed, glucose, inflammation, lipid, meta‐analysis

## Abstract

This meta‐analysis aimed to evaluate the effects of flaxseed supplementation on weight loss, lipid profiles, high‐sensitivity C‐reactive protein (hs‐CRP), and glucose levels in patients with coronary artery disease (CAD). A systematic search was performed using various online databases, including Scopus, PubMed, Web of Science, EMBASE, and Cochrane Library, to identify relevant randomized controlled trials (RCTs) until June 2023. To evaluate heterogeneity among the selected studies, the *Q*‐test and *I* ^2^ statistics were employed. Data were combined using either a fixed‐ or random‐effects model and presented as a weighted mean difference (WMD) with a 95% confidence interval (CI). Of the 428 citations, six RCTs were included. The pooled results did not show significant changes in the WMD of lipid factors (high‐density lipoprotein cholesterol, triglycerides (TG), low‐density lipoprotein cholesterol, and total cholesterol) following flaxseed intake. However, after performing a sensitivity analysis to determine the source of heterogeneity, flaxseed supplementation resulted in a significant decrease in TG levels (WMD = −18.39 mg/dL; 95% CI: −35.02, −1.75). Moreover, no significant differences were observed in either weight or BMI following flaxseed intake. However, the circulating levels of fasting blood glucose (WMD = −8.35 mg/dL; 95% CI: −15.01, −1.69, *p* = .01) and hs‐CRP (WMD = −1.35 mg/L; 95% CI: −1.93, −0.77, *p* < .01) significantly decreased after the intervention. Flaxseed supplementation was associated with lowering FBS, hs‐CRP, and TG levels but did not affect weight loss parameters and other lipid markers in CAD.

## Graphical abstract

This meta‐analysis assessed the efficacy of flaxseed supplementation in improving anthropometric, glycemic, lipid, and inflammatory indices in individuals with coronary artery disease (CAD). Pooled data showed that 10–24 weeks of flaxseed or flaxseed oil supplementation led to a statistically significant decrease in glucose, high‐sensitivity C‐reactive protein, and triglyceride levels in individuals with CAD.

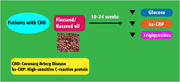

## 1 INTRODUCTION

Cardiovascular diseases (CVDs) continue to be a prominent cause of global mortality despite significant endeavors towards risk factor management and treatment improvement.^1^, ^2^ Therefore, CVD prevention is a significant public health concern and has wide‐ranging effects on the economy and healthcare system.^3^, ^4^ Moreover, developing countries face high rates of conventional risk factors for coronary artery disease (CAD), such as abdominal obesity and low physical activity.^5^, ^6^ Associations between inflammatory markers, lipid profiles, anthropometric indices, and CAD have been extensively studied.^7^, ^8^ Recent guidelines have placed great emphasis on reducing visceral fat and controlling dyslipidemia and blood pressure.^9^ Innovative nutritional strategies, including functional foods and herbal medicines have been developed.^10^, ^11^, ^12^ These alternative therapies are gaining popularity as individuals seek natural and safe approaches for disease prevention or to mitigate CAD risk.^12^, ^13^

Previous studies have shown different effects of herbal medicine on lipid and glycemic profiles and inflammatory parameters in CVDs.^14^, ^15^, ^16^ Flaxseed (*Linum usitassimum*) is a widely used herbal medicine that significantly affects multiple CVD risk factors.^17^ This is mainly because flaxseed contains a high amount of α‐linolenic acid (ALA) (22%), phytoestrogen, phenolic compounds,^18^ and lignans (0.2–13.3 mg/g flaxseed).^19^, ^20^ Furthermore, flaxseed serves as a highly beneficial dietary means of obtaining protein (28%–30%), vitamins, minerals, and a significant quantity of dietary fiber, weighing 28% of its total mass, with one‐third being soluble fiber.^21^, ^22^ Promising effects on lipid and glucose metabolism have been observed in patients with metabolic diseases who consumed flaxseed oil. In a study by Soleimani et al.^23^ the addition of flaxseed oil to the diet resulted in a significant decline in insulin resistance among patients with diabetic foot ulcer.^22^ Conversely, another study revealed a lack of evidence supporting the beneficial impact of flaxseed oil supplementation on glycemic control and lipid profiles among individuals with diabetes.^24^ Existing information regarding the association between flaxseed and inflammation is inconclusive and lacks agreement among researchers.^22^

Numerous studies have been conducted on flaxseed, but the results have been inconsistent with respect to its effects on glycemic and lipid profiles, weight loss, and inflammatory markers. Some studies have found positive effects, while others have not. Because of these inconsistent findings and the absence of a comprehensive meta‐analysis specifically focusing on patients with CAD, we aimed to incorporate all existing randomized controlled trials (RCTs) to assess the efficacy of flaxseed/flaxseed oil supplementation on glycemic and lipid profiles, body weight loss, and high‐sensitivity C‐reactive protein (hs‐CRP) in patients with CAD.

## 2 METHODS

### 2.1 Search and studies section

The current meta‐analysis was conducted in accordance with the Preferred Reporting Items for Systematic Reviews and Meta‐analysis guidelines (Table S1). Clinical trials published from the beginning to June 2023 were identified using a systematic search of online resources, including PubMed/Medline, Scopus, Embase, Web of Science, and Cochrane Library. We used the Patient, Intervention, Comparison Group, Outcome, and Study Design framework to identify studies that met our criteria, including RCTs with either a crossover or parallel design among patients with CADs, reported the effects of flaxseed consumption on weight loss, lipid profiles, glucose, and hs‐CRP as the primary outcomes in both the intervention and placebo groups, and were published in the English language without a time restriction. The following medical subject headings, subject terms, and keywords were applied to retrieve potentially relevant articles: (“flaxseed oil” OR “flaxseed” OR “linseed” OR “lignin” OR “Linum sitatissimum” OR “L. usitatissimum” AND “supplementation” OR “intake” OR “administration”) and (“CAD” OR “coronary artery disease” OR “coronary heart disease” OR “coronary disease” “OR “CHD” OR “coronary arteriosclerosis” OR “coronary arteriosclerosis”) and (“randomized controlled trial” OR “randomized clinical trial” OR “RCT” OR “controlled clinical trial” OR “randomized” OR “intervention studies” OR “controlled trial” OR “random*” OR “trial” OR “clinical trial”). The reference lists of the selected articles and previous reviews were manually checked to increase the sensitivity of our search strategy.

### 2.2 Inclusion and exclusion criteria

To incorporate RCTs into the study, the authors established certain criteria for inclusion and exclusion. (1) Articles with either a crossover or parallel design; (2) human clinical trials that investigated the effects of flaxseed/flaxseed oil on glucose and lipid profiles, weight loss, and inflammation biomarkers in patients with CAD, and sufficient data reported on mean changes of at least one of the studied outcomes with standard error, standard deviation (SD), or related 95% confidence interval (CI) at baseline and the end of intervention in both groups. Nonclinical studies, trials without a control group, and trials with insufficient information on the intervention and control groups were excluded.

### 2.3 Data extraction

Two authors (M. A. and M. M.) independently extracted the following items from the eligible articles: first author's name, study location, publication year, main features of participants, sample size of participants in the flaxseed oil and placebo groups, duration and dosage of intervention, study type, intervention type, placebo type, baseline dietary intake of energy, protein, carbohydrate, and total fat in the intervention and placebo groups; the mean and SD for fasting blood glucose (FBS), lipid profiles (total cholesterol [TC], low‐density lipoprotein cholesterol [LDL‐c], triglycerides [TG], and high‐density lipoprotein cholesterol [HDL‐c]), weight loss (weight and body mass index [BMI]), and inflammatory biomarkers (hs‐CRP) at baseline and the end of intervention in both the intervention and placebo groups. We only considered the outcomes in at least three clinical trials. Any discrepancies were resolved through discussion with a third author (M. R.).

### 2.4 Risk of bias (RoB) assessment

**Figure 1:**
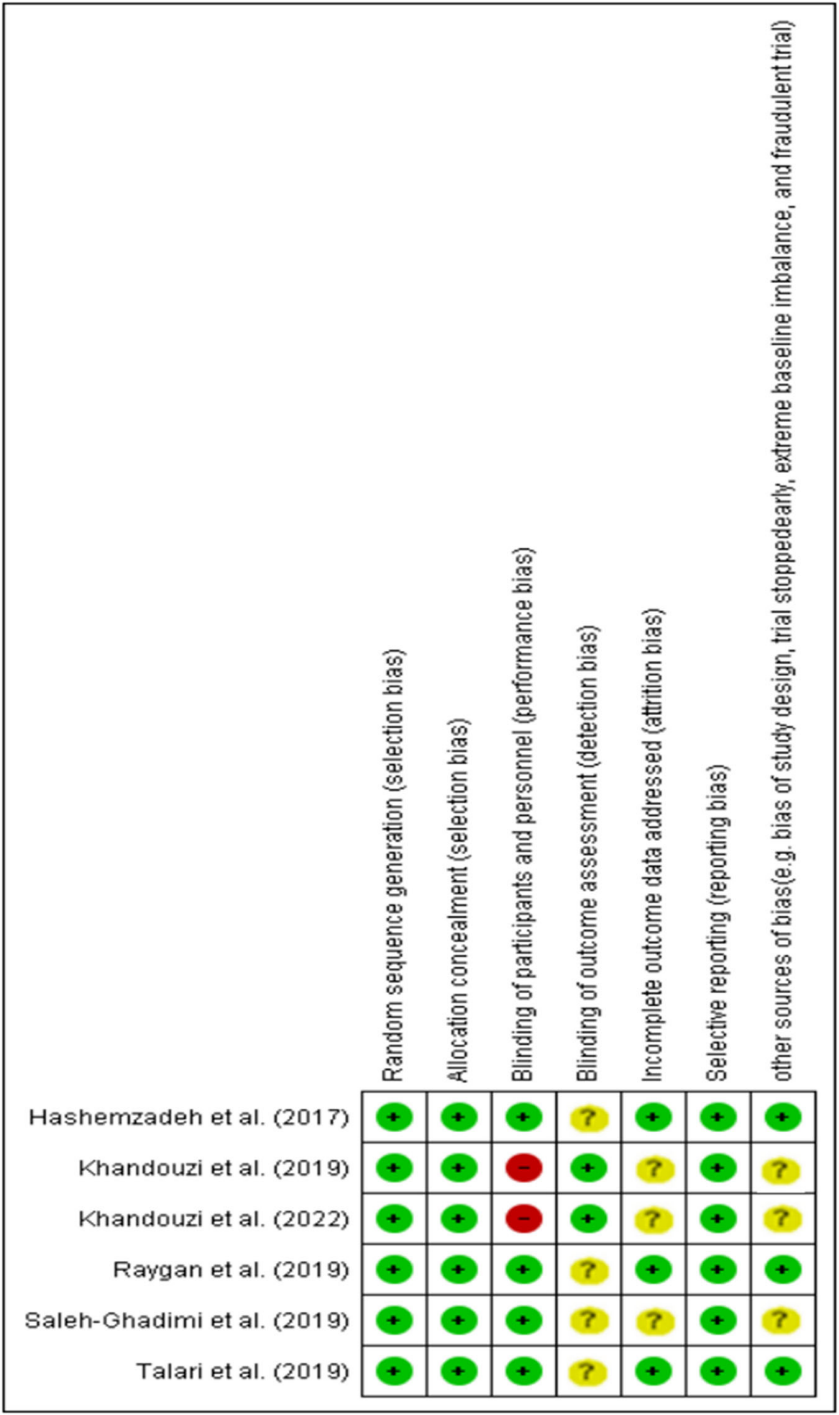
Risk of bias assessment result using the Cochrane RoB tool.

Applying the Cochrane RoB tool, the quality of the included articles was systematically as described elsewhere.^25^ Finally, as shown in Figure 1, the RoB in each trial was judged as low (green), high (red), or unclear (yellow).

### 2.5 Certainty of evidence assessment

The evaluation of the evidence for outcomes was carried out utilizing the modified Grading of Recommendations Assessment, Development, and Evaluation (GRADE) approach. This process was led by two independent investigators, O. K. and M. A., who evaluated the certainty of the body of evidence based on criteria such as the presence of RoB, indirectness, inconsistency, imprecision, and evidence of publication bias.

### 2.6 Statistical analysis

STATA 16.0 (Stata Corp.) and Review Manager 5.3 (Cochrane Collaboration) were used for all the statistical analyses. To quantify the effects of flaxseed oil on the changes in the following outcomes, data were pooled and the weighted mean difference (WMD) with 95% CI was utilized. (1) FBS, (2) TG, (3) TC, (4) LDL‐c, (5) HDL‐c, (6, 7) weight, (8) BMI, and (9) hs‐CRP. The effect size (ES) was determined using the mean change (SD). The DerSimoniane‐Laird technique with a random‐effects model was used for the ES meta‐analysis. The existence of significant interstudy heterogeneity among trials was assessed using Cochran's *Q* test (*p* < .1) and *I* ^2^ test (>50%). Sensitivity analysis was carried out using the leave‐one‐out technique to determine the source of heterogeneity after removing the trials individually. Egger's linear regression tests and funnel plots were applied to evaluate evidence of potential publication bias across studies.

## 3 RESULTS

### 3.1 Search results and characteristics of included trails

**Figure 2:**
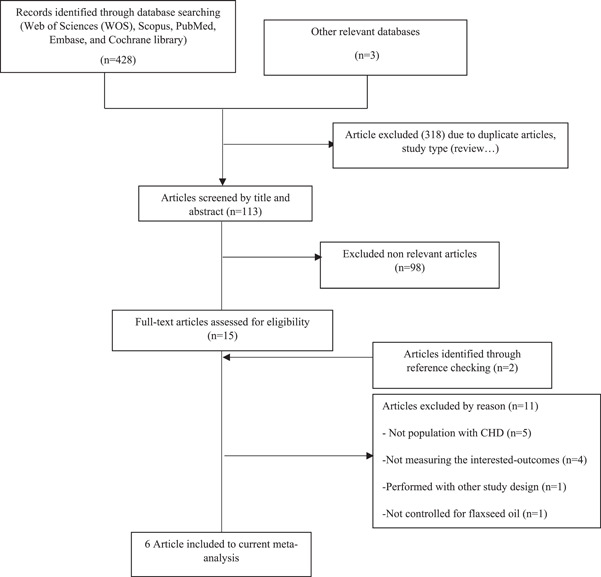
Flowchart of study identification and selection process.

The literature search yielded 531 reports. After removing duplicate articles (*n* = 318), 420 articles were left, 113 of which were disqualified based on their titles and abstracts. Finally, the full texts of 17 pertinent papers were retrieved for evaluation in accordance with the inclusion eligibility criteria. Eleven of these were omitted for the following reasons: not measuring the outcomes of interest (*n* = 4), performed with other study designs (*n* = 1), not population with CAD (*n* = 5), and not controlled for flaxseed/flaxseed oil (*n* = 1). Finally, six clinical trials^26^, ^27^, ^28^, ^29^, ^30^, ^31^ that were published between 2017 and 2022 were selected for the current meta‐analysis. Figure 2 shows the step‐by‐step process used to identify and select included articles.

Overall, 309 participants were randomized and completed the study period, of which 153 were randomly allocated to the flaxseed supplementation group and 156 to the placebo group in the six included clinical trials. FBS and hs‐CRP levels were investigated in three trials, lipid profiles in four, and weight and BMI in five. The intervention lasted between 10 and 24 weeks, and all the included trials used a parallel study design. Table 1 summarizes the general features of the six trials.

**Table 1:** Characteristics of the six selected clinical trials.

Author (publication year)	Study location	Sample size (intervention/placebo)	Mean age (intervention/placebo)	Gender in intervention, placebo (male/female)	Intervention type	Duration (weeks)	Participants	Dietary intakes Energy (kcal/d)	Dietary intakes of carbohydrate (g/d)	Protein (g/d)	Outcomes
Saleh‐Ghadimi et al. (2019)^25^, ^26^	Iran	21/19	55.67 ± 6.9, 54.80 ± 7.8	19/2, 17/2	200 mL of 1.5% fat milk + flaxseed oil (5 g)	10	CAD	2125.88 ± 280.19, 2099.95 ± 296.42	292.31 ± 38.53, 288.74 ± 40.76	79.73 ± 10.51, 78.75 ± 11.17	FBS, TG, TC, LDL‐c, HDL‐c, weight, BMI
Raygan et al. (2019)^27^	Iran	30/30	64.1 ± 9.3, 64.6 ± 9.1	14/16, 13/17	Flaxseed oil (1000 mg)	12	T2DM and CHD	2311 ± 261, 2371 ± 249	54.2 ± 6.9, 54.4 ± 4.6 (%)	15.0 ± 3.1, 14.3 ± 2.8 (%)	FBS, TG, TC, LDL‐c, HDL‐c, weight, BMI, hs‐CRP
Talari et al. (2019)^28^	Iran	30/31	67.3 ± 8.6, 66.4 ± 9.3	8/22, 9/22	50 000 IU vitamin D supplements every 2 weeks + 2 × 1000 mg/d n‐3 fatty acids from flaxseed oil	24	T2DM and CHD	–	–	–	FBS, TG, TC, LDL‐c, HDL‐c, weight, BMI, hs‐CRP
Khandouzi et al. (2019)^29^	Iran	21/23	56.67 ± 7.44, 56.22 ± 9.02	16/5, 21/2	Flaxseed (30 g/daily)	12	CAD	1777.67 ± 168.02, 1802.87 ± 150.82	243.00 ± 21.01, 246.91 ± 20.61	73.33 ± 10.59, 73.91 ± 9.29	Weight, BMI, hs‐CRP
Khandouzi et al. (2022)^30^	Iran	21/23	56.67 ± 7.44, 56.22 ± 9.02	16/5, 21/3	Flaxseed (30 g/daily)	12	CAD	1777.67 ± 168.02, 1802.87 ± 150.83	243.00 ± 21.01, 246.91 ± 20.62	73.33 ± 10.59, 73.91 ± 9.30	TG, TC, LDL‐c, HDL‐c
Hashemzadeh et al. (2017)^31^	Iran	30/30	59.2 ± 11.1, 60.0 ± 13.5	NR	Flaxseed oil (1000 mg twice a day)	12	T2DM and CHD	2230.5 ± 184.9, 2263.7 ± 198.1	306.3 ± 41.5, 319.0 ± 42.7	80.4 ± 16.5, 79.1 ± 16.4	Weight, BMI

Abbreviations: ALA, α‐linolenic acid; BMI, body mass index; CAD, coronary artery disease; CHD, congenital heart disease; FBS, fasting blood glucose; HDL‐c, high‐density lipoprotein‐cholesterol; hs‐CRP, high sensitive C‐reactive protein; LDL‐c, low‐density lipoprotein‐cholesterol; NR, not reported; T2DM, type 2 diabetes mellitus; TC, total cholesterol; TG, triglycerides.

### 3.2 Meta‐analysis results

#### 3.2.1 Impact of flaxseed supplementation on FBS

With respect to the effects of flaxseed supplementation on FBS levels in patients with CAD, a meta‐analysis using a random‐effects model of three included trials revealed a significant decrease in this parameter following flaxseed treatment (WMD = −8.35 mg/dL; 95% CI: −15.01, −1.69, *p* = .01), following flaxseed treatment, with an insignificant statistical heterogeneity among trials (*p* = .72, *I* ^2^ = 0.0%) (Figure 3A).

Figure 3(A–H) The effects of flaxseed supplementation on (A) FBS, (B) TG, (C) TC, (D) LDL‐cholesterol, (E) HDL‐cholesterol, (F) weight, (G) BMI, (H) C‐reactive protein in patients with CAD. BMI, body mass index; CAD, coronary artery disease; CI, confidence interval; FBS, fasting blood glucose; HDL, high‐density lipoprotein; LDL, low‐density lipoproteinTC, total‐cholesterol; TG, triglycerides; WMD, weighted mean difference.

**Figure:**
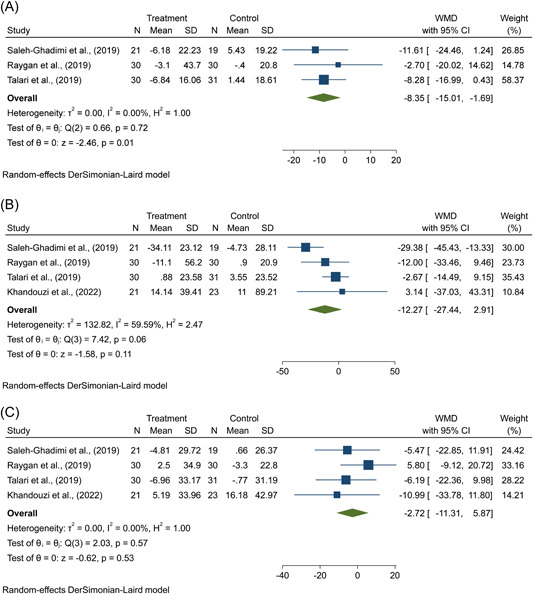

**Figure:**
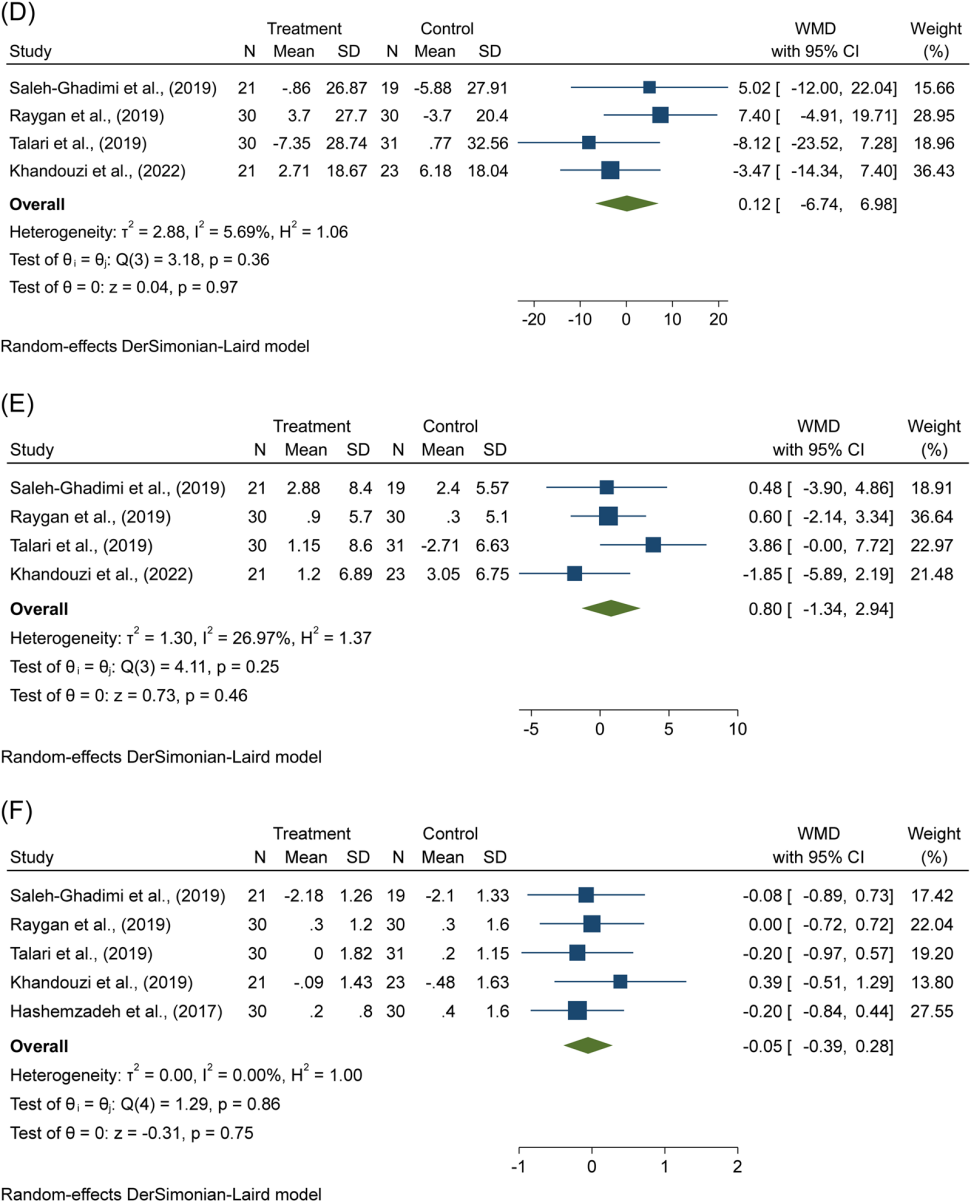

**Figure:**
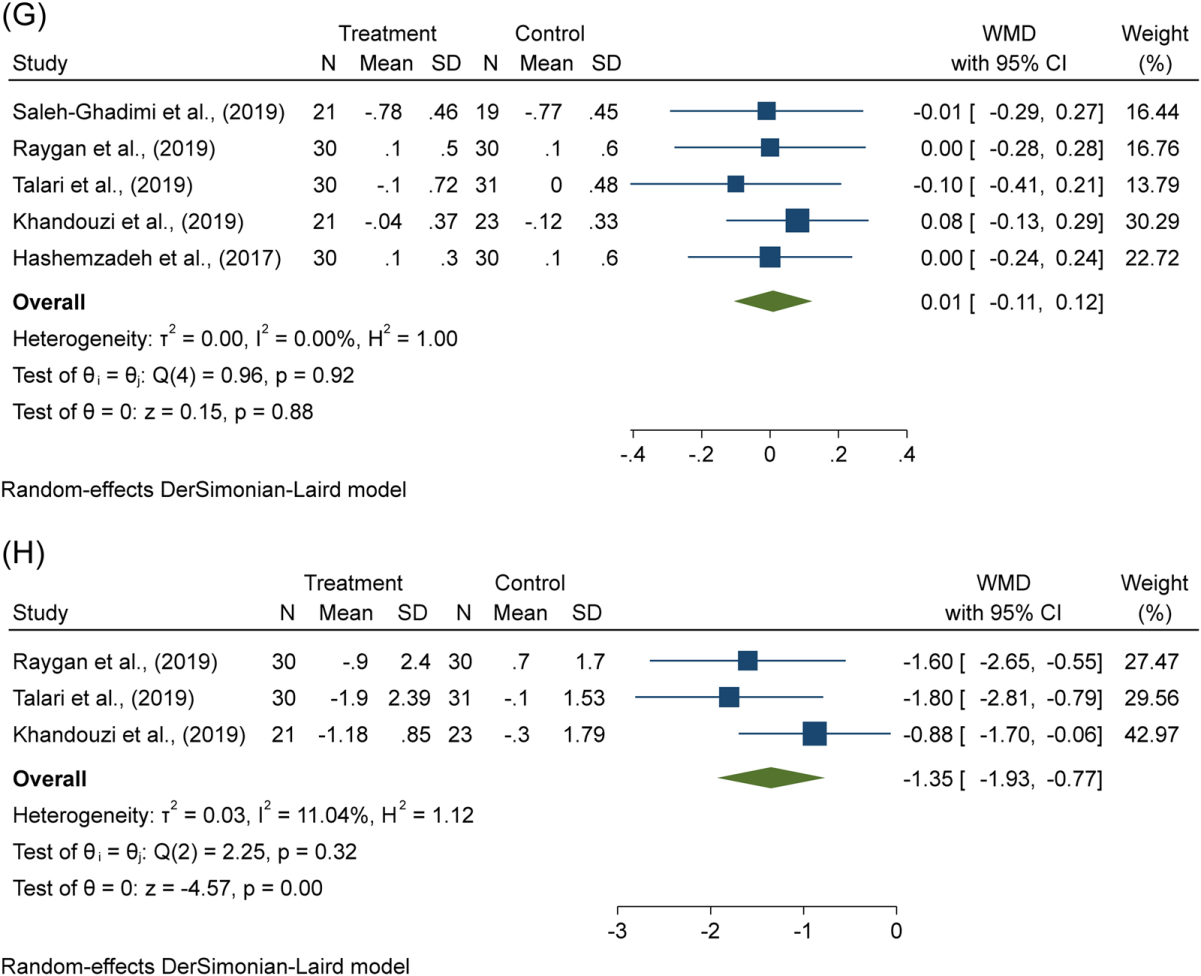

#### 3.2.2 Impact of flaxseed supplementation on lipid profiles

Flaxseed supplementation did not have a noticeable impact on TG (WMD = −12.27 mg/dL; 95% CI: −27.44, 2.91, *p* = .11; *I* ^2^ = 59.59%), TC (WMD = −2.72 mg/dL; 95% CI: −11.31, 5.87, *p* = .53; *I* ^2^ = 0.0%), LDL‐c (WMD = 0.12 mg/dL; 95% CI: −6.74, 6.98, *p* = .97; *I* ^2^ = 5.69%), and HDL‐c levels (WMD = 0.80 mg/dL; 95% CI: −1.34, 2.94, *p* = .46; *I* ^2^ = 26.97%) according to the four‐trial random‐effects meta‐analysis compared with controls in patients with CAD (Figure 3B–E).

Based on the significant heterogeneity in TG levels between studies (*p* = .06, *I* ^2^ = 59.59%), we conducted a sensitivity analysis to identify the source of heterogeneity by excluding each study individually. After removing the study conducted by Talari et al.,^28^ we observed a reduction in interstudy heterogeneity to *I* ^2^ = 37%. Moreover, flaxseed supplementation had a noticeable impact on TG levels (WMD = −18.39 mg/dL; 95% CI: −35.02, −1.75) after excluding this particular trial (Figure S1).

#### 3.2.3 Impact of flaxseed supplementation on weight loss

There was no significant effect of flaxseed supplementation on weight (WMD = −0.05 kg; 95% CI: −0.39, 0.28, *p* = .75; *I* ^2^ = 0.0%) and BMI (WMD = 0.01 kg/m^2^; 95% CI: −0.11, 0.12, *p* = .88; *I* ^2^ = 0.0%) according to the pooled results using the random‐effects model from five trials in patients with CAD (Figure 3F,G).

#### 3.2.4 Impact of flaxseed supplementation on hs‐CRP

As shown in Figure 3H, the random‐effects meta‐analysis using a random‐effects model of three trials showed that there was a significant reduction in hs‐CRP levels (WMD = −1.35 mg/L; 95% CI: −1.93, −0.77, *p* < .01; *I* ^2^ = 11.04%), following supplementation with flaxseed products.

### 3.3 Publication bias

There was no significant indication of publication bias in Egger's linear regression test for trials examining the impact of flaxseed supplementation on FBS (*p* = .73), TG (*p* = .95), TC (*p* = .23), HDL‐c (*p* = .98), LDL‐c (*p* = .94), weight (*p* = .14), and hs‐CRP (*p* = .21) levels. With respect to BMI, we found significant publication bias (*p* = .03). However, after conducting a trim‐and‐fill sensitivity analysis including censored studies, we found that the pooled WMD for BMI remained unchanged. Visual examination of the funnel plots, shown in Figure S2, found no evidence of possible publication bias.

### 3.4 Certainty of evidence

Table 2 presents the certainty of the evidence pertaining to the outcomes obtained by employing a revised GRADE method. The certainty of the evidence was deemed to be low for the three outcomes and very low for the five other outcomes that were taken into account. The primary factors that resulted in downgrading the certainty of the evidence included bias, indirectness, and imprecision.

**Table 2:** Summary of findings using revised GRADE profile.

Pooled effect MD (95% CI)	No. of studies	Study design	Risk of bias	Inconsistency	Indirectness	Imprecision	Publication bias	Certainty of evidence
Studied outcomes								
FBS (mg/dL)								
−8.35 (−15.01, −1.69)	3	Clinical trial	0	0	−1^a^	−2^b^	0	**+ − − −**(Very low)
TG (mg/dL)								
−12.27 (−27.44, 2.91)	4	Clinical trial	−1	−1^c^	−1	−1^d^	0	**+ − − −**(Very low)
TC (mg/dL)								
−2.72 (−11.31, 5.87)	4	Clinical trial	−1	0	−1	−1	0	**+ − − −**(Very low)
LDL‐c (mg/dL)								
0.12 (−6.74, 6.98)	4	Clinical trial	−1	0	0	−1	0	**+ + − −**(Low)
HDL‐c (mg/dL)								
0.80 (−1.34, 2.94)	4	Clinical trial	−1	0	−1	−1	0	**+ − − −**(Very low)
Weight (kg)								
−0.05 (−0.39, 0.28)	5	Clinical trial	−1	0	−1	0	0	**+ + − −**(Low)
BMI (kg/m^2^)								
0.01 (−0.11, 0.12)	5	Clinical trial	−1	0	0	0	−1	**+ + − −**(Low)
Hs‐CRP (mg/L)								
−1.35 (−1.93, −0.77)	3	Clinical trial	−1	0	−1	−2	0	**+ − − −**(Very low)

*Note*: ++−− indicates the certainty of evidence.

Abbreviations: CI, confidence interval; BMI, body mass index; FBS, fasting blood glucose; GRADE, grades of recommendation, assessment, development, and evaluation; HDL‐c, high‐density lipoprotein‐cholesterol; hs‐CRP, high sensitive C‐reactive protein; LDL‐c, low‐density lipoprotein‐cholesterol; MD, mean difference; TG, triglycerides; TC, total cholesterol.

^a^ One level was demoted due to the significant degree of indirectness.

^b^ One level was demoted due to the substantial level of imprecision.

^c^ One level was demoted due to the inconsistency (*I* ^2^) exceeding 50%.

^d^ Two levels were demoted due to the number of studies being less than three and a considerable level of imprecision.

## 4 DISCUSSION

The present meta‐analysis investigated the efficacy of flaxseed on anthropometric, glycemic, lipid, and inflammatory indices in individuals with CAD. The pooled results indicated that 10–24 weeks of supplementation with flaxseed or flaxseed oil had considerable therapeutic effects on glucose, hs‐CRP, and TG levels in individuals with CAD.

Dyslipidemia is a condition that increases the risk for atherosclerosis and CAD, resulting in numerous fatalities globally.^32^ We observed a notable decrease in circulating TG levels, suggesting the potential cardioprotective properties of flaxseed oil. However, no alterations were observed in the other lipid parameters. Previous studies have presented varying outcomes regarding the impact of flaxseed supplementation on lipid profiles. A comprehensive meta‐analysis by Hadi et al.,^4^ including 62 RCTs, showed that flaxseed supplementation may improve blood levels of TC, TG, and LDL‐C, but not HDL‐c, in various healthy and unhealthy subjects, indicating that it may delay the development of heart disease. In addition, Masjedi et al.^33^ also conducted a meta‐analysis to determine the impact of flaxseed on blood levels of lipid markers in healthy and dyslipidemic individuals, demonstrating the same results as those obtained by Hadi et al.^4^ Similarly, Dodin et al.^34^ conducted a study that found that the daily consumption of 40 g of flaxseed led to improved lipid factor levels in healthy menopausal women after 12 months. However, another study indicated that the lipid profile did not exhibit any reduction after a 6‐week period of consuming 15 mL/day of ALA flaxseed oil when compared to the consumption of olive oil during the same duration.^35^ Interestingly, another RCT also demonstrated the opposite effect of flaxseed supplementation on TG (an increase of 12.0% from baseline to 8 weeks following intervention) in elderly subjects.^36^ The changes in lipid profiles observed in this study may be attributed to several factors, including the consumption of flaxseed or flaxseed oil, variations in absorption efficiency among participants, adherence to the prescribed diet, and individual characteristics such as genetic background, lifestyle choices, age, and sex.^37^ Moreover, it is worth noting that the absence of a substantial impact of flaxseed oil on lipid profiles, as observed in the current meta‐analysis, may be partially attributed to the majority of participants having normal initial levels of these measures. This could potentially be attributed to prior statin therapy administration. The positive effects of flaxseed oil on lipid metabolism may be related to its ability to enhance lipid balance within the adipose tissue‐liver axis and promote fatty acid β‐oxidation.^38^ Furthermore, flaxseed oil has been shown to reduce lipogenesis, resulting in decreased TG levels.^39^

The antidyslipidemic effects of flaxseed have been attributed to multiple mechanisms. Flaxseed, a rich source of ALA and secoisolariciresinol diglucoside, has been demonstrated to have antidyslipidemic properties.^40^ ALA undergoes conversion into long‐chain omega‐3 fatty acids and promotes cholesterol efflux from the macrophages. Its high polyphenol content may also contribute to its hypolipaemic effects. The high fiber content of flaxseed exerts antidyslipidemic effects by promoting satiety, reducing caloric intake, and reducing food transit time.^4^, ^41^

Askarpour et al.^42^ investigated the effects of flaxseed supplementation on proinflammatory markers and endothelial function in an overall population. A significant decrease in circulating CRP levels was observed in 35 RCTs, which is consistent with the findings of this study. In fact, a subgroup analysis conducted by the researchers showed that flaxseed significantly lowered CRP levels in unhealthy or overweight subjects. These effects were observed in RCTs that were given whole flaxseed or lignan supplements for more than 12 weeks. In contrast, another meta‐analysis did not find any significant beneficial effects of flaxseed or its derived products (such as lignans or flaxseed oil) on CRP levels in the general population.^21^ There have been several reported mechanisms to account for flaxseeds' ability to reduce inflammation. One such mechanism is the presence of ALA, a PUFA‐omega‐3 fatty acid found abundantly in flaxseeds. Long‐chain omega‐3 fatty acids, which are recognized to have anti‐inflammatory qualities, can be produced from ALA.^40^ ALA may not exert the same impacts on inflammation. Furthermore, the amount of fiber present in flaxseed may contribute to its ability to protect against inflammation by producing short‐chain fatty acids, such as propionate, acetate, and butyrate, which can reduce the activity of certain proinflammatory cytokines in adipose tissue.^21^ The discrepancies in the findings could be attributed to other factors, such as dietary components, lifestyle choices, or the genetic factors of the participants. Furthermore, various bioactive components found in flaxseed oil and flaxseed, as well as differences in production methods, compound stability, storage conditions, and the use of whole flaxseed or its derivatives, could affect bioavailability and subsequently affect the response of adhesion molecules and inflammatory markers.^20^, ^42^

The results of the study indicated that there was no substantial impact on body weight and BMI between the two groups when consuming flaxseed. Pan et al.^43^ conducted a meta‐analysis that revealed that whole flaxseed, rather than flaxseed oil, had a notable impact on weight and BMI reduction. This effect is likely due to the abundant fiber content in flaxseed, which aids in controlling energy intake and enhancing satiety. However, the outcomes did not demonstrate statistical significance in investigations in which flaxseed oil supplementation (four trials) was administered instead of flaxseed (one trial). The specific mechanism by which flaxseed exerts its antiobesity effects remains unclear. Current evidence suggests that the bioactive components of flaxseed oil, particularly ALA, may play a role in reducing obesity.^44^ Furthermore, flaxseed oil is rich in various unsaturated fatty acids such as linoleic and eicosadienoic acids, along with ALA, all of which possess antiobesity properties.^45^ The variations in the obtained results could be explained by several factors, including variations in the duration and dosage of the intervention, type of flaxseed oil and ALA sources used, differences in dietary intake, clinical condition of the participants, and levels of physical activity.

The impacts of flaxseed and flaxseed oil on FBS have been evaluated in a restricted range of studies.^26^, ^35^, ^46^ A meta‐analysis found that while whole flaxseed had a significant impact on improving glycemic control in the general population, lignan extract and flaxseed oil did not have the same effect.^46^ This finding was consistent with that of the present meta‐analysis. However, other RCTs have not demonstrated a significant reduction in FBS levels following flaxseed supplementation. More extensive trials are necessary to fully understand the effects of flaxseed on the glycemic profile of individuals with CAD.

Although reports on the positive impacts of flaxseed on human health have been documented, concerns exist within certain populations, particularly pregnant women. However, animal studies have demonstrated that exposure to estrogen during the neonatal stage can lead to a decrease in sperm production. Therefore, the consumption of flaxseed, especially over a prolonged period, should be approached with caution by pregnant women and men of reproductive age, particularly in cases of chronic consumption.^47^

This study has several limitations that require attention. Initially, the absence of data in the included RCTs prevented the measurement of other well‐known risk factors for coronary heart disease, such as inflammatory markers. It is also important to consider the impact of confounding parameters, such as lifestyle factors and genetic background, on the effectiveness of flaxseed supplements, as well as their formulation. The relatively short intervention period and small sample size are acknowledged as the limitations of this study. However, it is worth noting that this is the first meta‐analysis to assess the effects of flaxseed administration on FBS, hs‐CRP, body weight loss, and lipid parameters in patients with CAD. Furthermore, efforts were made to minimize bias and heterogeneity in the review process through a comprehensive literature search and sensitivity analysis.

## 5 CONCLUSION

The findings showed that flaxseed intake had a positive impact on the reduction of TG, FBS, and hs‐CRP levels in patients with CAD. This finding suggests that flaxseed consumption may significantly mitigate the overall risk factors associated with CAD. Future research should focus on designing large, long‐term trials that minimize bias and adhere to current reporting criteria for clinical trials. Additionally, it is important to assess whether any positive effects are sustained over time. Further RCTs are needed to investigate the effects of flaxseed supplementation on other cardiovascular consequences, such as stroke and myocardial infarction.

## AUTHOR CONTRIBUTIONS

**Hamid R. Sabet**: Project administration; methodology; review and editing. **Mohammad Ahmadi**: Writing—original draft; methodology; software. **Mehdi Akrami**: Writing—original draft; visualization; investigation. **Mahsa Motamed**: Writing—original draft; software; visualization. **Omid Keshavarzian**: Writing—original draft; resources. **Mozhan Abdollahi**: Validation; resources; writing—original draft. **Mehdi Rezaei**: Conceptualization; supervision; methodology; review and editing. **Hamed Akbari**: Conceptualization; project administration; supervision; review and editing.

## CONFLICT OF INTEREST STATEMENT

The authors declare no conflicts of interest.

## Supporting information

Supporting information.

Supporting information.

## Data Availability

The data that support the findings of this study are available from the corresponding author upon reasonable request.
