# Supplementary material for: Effects of flaxseed supplementation on weight loss, lipid profiles, glucose, and high‐sensitivity C‐reactive protein in patients with coronary artery disease: A systematic review and meta‐analysis of randomized controlled trials
Source: Clin Cardiol. 2024 Jan 16;47(1):e24211. doi: 10.1002/clc.24211 (PMC10790321; doi:10.1002/clc.24211)
Supplement: Supplementary file 2 — Supporting information. [file CLC-47-e24211-s002.docx]

**Supp2. Fig.1s.** Sensitivity analysis plot for TG level.

**Supp2. Fig. 2s**. Provides visual inspection of funnel plots on FBS (A), TG (B), TC (C), LDL-cholesterol (D), HDL-cholesterol (E), weight (F), BMI (G) C-reactive protein (H)

FBS (A)

TG (B)

TC (C)

LDL-cholesterol (D)

HDL-cholesterol (E)

Weight (F)

BMI (G)

C-reactive protein (H)
